# Intercontinental aeromedical transfers after decompressive craniectomy for severe traumatic brain injury: Systematic review and evidence-informed fitness-to-fly pathway

**DOI:** 10.1016/j.bas.2026.106278

**Published:** 2026-08-09

**Authors:** Deepak Gupta, Vishal Garg, Ritwik Kishore, Rajeev Sharma, Kushagra Pandey, Atin Kumar, Babita Gupta, Kamran Farooque, Anna Teresa Mazzeo

**Affiliations:** a Department of Neurosurgery, Jai Prakash Narayan Apex Trauma Centre (JPNATC), All India Institute of Medical Sciences (AIIMS), New Delhi, India; b Department of Radiodiagnosis and Interventional Radiology, Trauma Centre Division, Jai Prakash Narayan Apex Trauma Centre (JPNATC), All India Institute of Medical Sciences (AIIMS), New Delhi, India; c Department of Anaesthesiology, Pain Medicine and Critical Care, Jai Prakash Narayan Apex Trauma Centre (JPNATC), All India Institute of Medical Sciences (AIIMS), New Delhi, India; d Department of Orthopaedics, Jai Prakash Narayan Apex Trauma Centre (JPNATC), All India Institute of Medical Sciences (AIIMS), New Delhi, India; e Anesthesia and Intensive Care, Department of Human Pathology, University of Messina, Messina, Italy

**Keywords:** Aeromedical transfer, Decompressive craniectomy, Traumatic brain injury, Pneumocephalus, Fitness to fly, Systematic review

## Abstract

**Background:**

Traumatic brain injury (TBI) sustained abroad creates major clinical, logistical, and psychosocial challenges. Guidance on the timing and safety of aeromedical repatriation after cranial surgery remains inconsistent and largely unvalidated.

**Case description:**

A 26-year-old foreign national presented unconscious after a hotel-balcony fall. Computed tomography showed a right frontotemporoparietal acute subdural haematoma with bilateral basifrontal contusions.

**Intervention and outcome:**

Following resuscitation, he underwent decompressive craniectomy and haematoma evacuation. After tracheostomy and neurocritical-care stabilization, multidisciplinary coordination was undertaken with his family, insurer, authorities, and receiving centre. Pre-flight assessment confirmed neurological and cardiopulmonary stability, secure airway and ventilation, and absence of pneumocephalus and pneumothorax. Three weeks after surgery, transfer by ventilator-equipped fixed-wing air ambulance was completed without reported complications.

**Systematic review:**

A PRISMA 2020-aligned focused review was conducted on aeromedical transfers for patients after decompressive craniectomy for severe TBI. Of 737 records identified, 58 full-text reports were assessed, and twenty-six primary studies were included in the narrative synthesis; eight professional guidance, consensus, and regulatory documents were synthesized separately. Evidence was heterogeneous, comprising mainly retrospective military cohorts, surveys, mechanistic models, and case reports. Secondary physiological insults during transport—not elapsed postoperative time alone—were the recurring risk. Evidence relating intracranial-air volume to deterioration was inconsistent, and no universally safe volume or validated post-craniectomy interval was identified.

**Conclusion:**

Long-distance repatriation after decompressive craniectomy may be feasible following structured multidisciplinary assessment, physiological optimisation, exclusion of trapped intracranial or thoracic air, and appropriate cabin-pressure and monitoring strategies. The proposed defer/conditional/ready pathway requires prospective validation.

## Highlights

• Fitness to fly decision after TBI or craniectomy is challenging and consensus is needed.
• Head and chest imaging should exclude pneumocephalus and pneumothorax.
• Intracranial-air cutoffs are model-derived, not stand-alone clearance gates.
• Cabin-pressure optimisation limits hypoxia and expansion of residual gas.
• A sequential PASS/FAIL pathway supports safe transcontinental repatriation.

## 1 Introduction

Aeromedical repatriation after decompressive craniectomy is uncommon but increasingly encountered in international trauma care. Cabin pressurization in fixed wing transport exposes vulnerable patients to hypobaric hypoxia and expansion of trapped gas, with potential consequences for intracranial pressure, pneumothorax, tracheostomy cuff pressure, ventilation, haemodynamic stability, equipment constraints and long periods outside a definitive care environment. These hazards are particularly important after severe traumatic brain injury (TBI) when hypotension, hypoxaemia, hypercarbia, seizures may cause secondary brain injury (Goodman et al., 2010; Fang et al., 2010; Dukes et al., 2013; Johannigman et al., 2015).

Published practice has often relied on elapsed time from TBI or surgery or proposed intracranial-air thresholds in neurotrauma patients requiring air transfer. However, the available thresholds arise mainly from models, small observational series, and professional opinion rather than prospective outcome validation (Andersson et al., 2003; Brändström et al., 2017; Lim et al., 2020; Bichsel et al., 2022; Donovan et al., 2008; Lindvall et al., 2008; Amato-Watkins et al., 2013; Mampre et al., 2026; Seth et al., 2009). We therefore report a successful intercontinental transfer after decompressive craniectomy, systematically review the relevant evidence, compare current recommendations, and propose a practical evidence-informed pathway regarding the timing, indications, contraindications, logistical considerations, and outcomes of aeromedical transfer after severe TBI and decompressive craniectomy. The case itself is not presented as proof of a novel intervention; its value is as an applied example within a transparent synthesis of a sparse and heterogeneous evidence base.

**Case Report**: We report a patient with severe TBI who underwent decompressive craniectomy (DC) in India and was subsequently transferred to a European country by air ambulance after structured neurocritical and aeromedical assessment. The case highlights practical decisions that health care workers and intensivists face when asked to certify fitness to fly after recent cranial surgery. We describe the structured assessment and successful intercontinental transfer of a patient after DC and propose a practical multidisciplinary pathway for evaluating fitness to fly after severe TBI.

A 26-year-old European citizen of Indian origin was brought to the neurosurgical emergency department after falling from the balcony of his hotel. On arrival, he was haemodynamically stable but unconscious, with a Glasgow Coma score of E1V1M5, consistent with severe TBI. Because of a threatened airway, he was intubated and mechanically ventilated. Primary survey revealed reduced breath sounds over the right hemithorax; suspected pneumothorax was treated with insertion of a right intercostal drain. There was no clinical evidence of maxillofacial or long-bone injury.

**Fig. 1:**
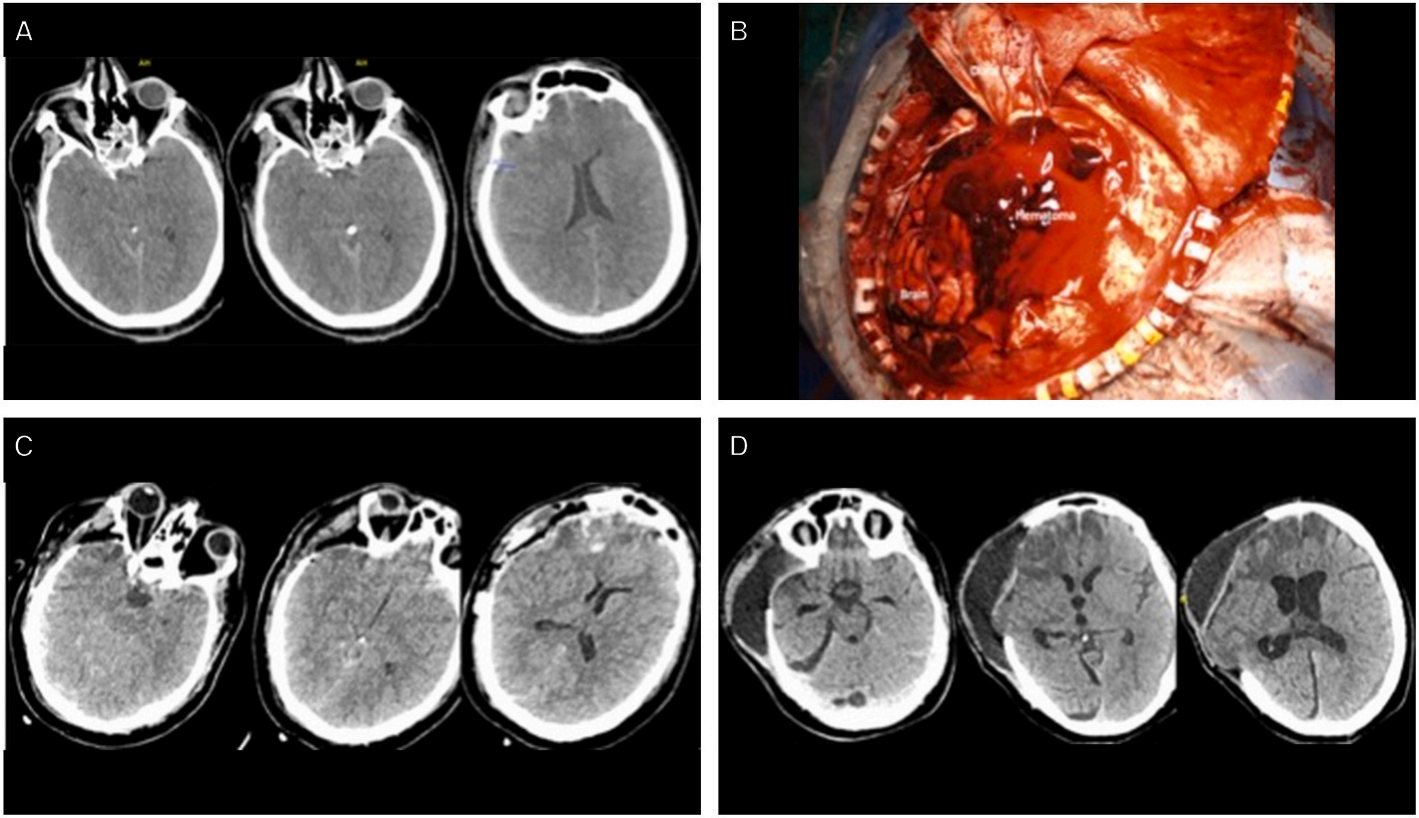
Serial neuroimaging and operative findings. (a) Admission non-contrast CT head showing right frontotemporoparietal acute subdural haematoma with cerebral edema, obliterated basal cisterns, and subarachnoid haemorrhage; Rotterdam CT score 5. (b) Intraoperative photograph after right frontotemporoparietal decompressive craniectomy showing swollen brain with overlying subdural haematoma. (c) Early postoperative bedside CT showing adequate decompressive craniectomy, resolving mass effect and midline shift, and evolving right basifrontal contusion. (d) CT head at 3 weeks before aeromedical transfer showing open basal cisterns, further resolution of mass effect, persistent craniectomy defect, and subgaleal hygroma.

Initial non-contrast computed tomography of the head demonstrated a right frontotemporoparietal acute subdural haematoma, diffuse subarachnoid haemorrhage involving the basal and perimesencephalic cisterns, cerebral edema, and mass effect; the Rotterdam CT score was 5. Emergency right frontotemporoparietal decompressive craniectomy and evacuation of the haematoma were performed. The dura was tense and bulging, and the underlying brain remained oedematous after haematoma evacuation. Expansile duraplasty was undertaken using autologous pericranium. Because of persistent cerebral swelling, the 15 × 12 cm bone flap was not replaced and was stored at −80°C in the institutional bone bank for subsequent autologous cranioplasty (Fig. 1a–c).

On postoperative day 2, chest radiography showed adequate lung expansion, allowing removal of the intercostal drain. The patient was managed in the neurotrauma intensive care unit according to standard severe TBI protocols. Owing to persistent impaired consciousness and the need for airway protection, percutaneous tracheostomy was performed on postoperative day 4. Enteral nutrition, deep-vein thrombosis prophylaxis, antiseizure medication, chest physiotherapy, and neurocritical monitoring were continued. Postoperative CT confirmed adequate decompression and resolution of mass effect and herniation. He was gradually weaned from ventilatory support and maintained an oxygen saturation of 94% on room air by postoperative day 12.

Two weeks after surgery, the family requested international repatriation. Transfer was considered high risk because of the recent DC, severe TBI, tracheostomy, previous pneumothorax, and potential hypoxaemia or expansion of trapped gas during altitude exposure. A structured neurocritical and aeromedical assessment therefore evaluated neurological stability, repeat cranial imaging, exclusion of pneumocephalus and thoracic air, respiratory reserve, haemodynamic stability, airway security, aspiration and seizure risk, cabin-pressure requirements, receiving-centre acceptance, and bone-flap transport logistics.

One day before transfer, the neurological examination was E2VTM5, without deterioration in motor response, new pupillary abnormality, focal deficit, or seizure during the preceding 24 h. Repeat CT showed no residual haematoma, hydrocephalus, pneumocephalus, or mass effect (Fig. 1d). Chest radiography and lung ultrasonography excluded pneumothorax and haemothorax. With the tracheostomy on room air, oxygen saturation was 96%. Arterial blood gas analysis showed pH 7.47, PaO_2_ 104 mm Hg, PaCO_2_ 40 mm Hg, bicarbonate 28 mmol/L, and lactate 0.8 mmol/L. Haemoglobin was 12 g/dL, and serum sodium, potassium, and glucose were within acceptable ranges. Cultures were sterile, viral screening was negative, and the surgical wound was healthy.

Before handover, tracheostomy position, patency, fixation, and cuff pressure were verified, with planned reassessment during ascent and descent. Suction equipment, bag-valve-mask ventilation, emergency airway supplies, medications, and two functioning intravenous lines accompanied the patient. Enteral feeding was withheld during transfer, and antiseizure medication was administered before departure.

Clinical records, imaging, operative details, and physiological data were shared with the receiving intensive care unit, which formally accepted the patient. The insurer arranged a ventilator-equipped air ambulance, and a fit-to-fly certificate specified cabin-pressure optimisation and continuous cardiorespiratory and airway monitoring. The autologous bone flap was released with institutional approval and transported at −20°C in sterile, labelled packaging with documentation confirming ownership, clinical necessity, and negative infectious-disease screening.

On postoperative day 20, the patient was transferred under medical supervision without reported in-flight complications. Continuous high-resolution in-flight physiological data were not available. At three months, caregiver-reported Glasgow outcome scale-Extended ( GOS-E) was 3 (lower severe disability).

**Systematic review**: Following successful intercontinental transfer after decompressive craniectomy in a patient with severe TBI, we conducted a systematic review on the relevant evidence, to compare current recommendations, and propose a practical evidence-informed pathways involving intercontinental aeromedical transfers for patients after DC for severe TBI.

## 2 Reporting framework and review questions

The review was reported with reference to PRISMA 2020 (Page et al., 2021). The research question was: in patients with severe TBI, recent intracranial surgery (including decompressive craniectomy), or pneumocephalus who undergo fixed-wing air transport, what evidence informs timing, indications, contraindications, physiological risks, mitigation, monitoring, and outcomes. No protocol was prospectively registered.

## 3 Information sources and search strategy

A comprehensive literature search was conducted in PubMed/MEDLINE, Embase, Scopus, and ClinicalTrials.gov on 24 July 2026. Searches were restricted to studies published in English. Search strategies were developed using controlled vocabulary and free-text terms related to traumatic brain injury/neurosurgical procedures, air travel or aeromedical transport, and pressure-related physiological effects. Two reviewers independently screened titles/abstracts and assessed full texts, with disagreements resolved through discussion. The full search strategies for all databases are provided in Supplementary Table 1 (Supplementary File S1).

## 4 Eligibility, selection, and data collection

Eligible reports involved human TBI, cranial surgery, or pneumocephalus during fixed-wing transport/commercial flight, or directly relevant models/surveys addressing cabin altitude, intracranial air, or postoperative clearance. Reviews and operational frameworks were used as contextual evidence but were not counted among the 26 primary studies. Reports limited to scene-to-hospital helicopter triage without a relevant in-flight physiological or postoperative question, non-neurological populations, animal studies, and commentaries without useable evidence were excluded.

Extracted fields were design, setting, sample size, neurological population, timing, cabin/altitude strategy, monitoring, secondary insults, clinical outcomes, and stated limitations. Because designs and outcomes were heterogeneous and several military publications used overlapping casualty cohorts, results were synthesized narratively and were not pooled. Methodological limitations were appraised by design: selection/confounding and incomplete charting for retrospective cohorts; response and consensus bias for surveys; publication bias for case reports; and indirectness for models. Formal certainty grading was not performed.

## 5 Systematic review results

### 5.1 Study selection and characteristics

**Fig. 2:**
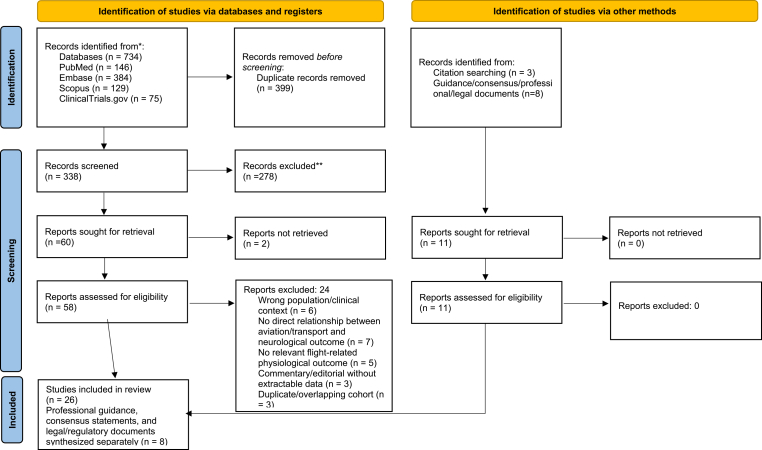
PRISMA 2020 flow diagram for the focused systematic review. Contextual professional guidance was synthesized separately from primary study selection.

The search identified 737 records, including 734 records from PubMed/MEDLINE, Embase, Scopus, and ClinicalTrials.gov, and three additional records identified through citation searching. After removal of 399 duplicate records, 338 records underwent title and abstract screening. Sixty reports were sought for full-text review; two could not be retrieved, leaving 58 reports assessed for eligibility. Twenty-four reports were excluded after full-text assessment due to reasons including wrong population or clinical context (n = 6), lack of relevant aviation or air transport exposure (n = 7), absence of useable flight-related outcomes (n = 5), commentary or non-data publications (n = 3), and duplicate or overlapping cohorts (n = 3). Twenty-six studies (clinical/research reports) plus eight guidance/regulatory documents were included in the narrative synthesis (Fig. 2). Eight professional guidance documents, consensus statements, and legal/regulatory documents were synthesized separately and were not counted as primary studies. Table 1 summarizes the most decision-relevant evidence reports for aeromedical transfer after severe TBI, cranial surgery, or pneumocephalus (Lindvall et al., 2008; Amato-Watkins et al., 2013; Mampre et al., 2026; Seth et al., 2009; Boyd et al., 2017; Maddry et al., 2020a, 2020b; Davis et al., 2022; Ng et al., 2023; Fang et al., 2010; Powell and Cramb, 2025; Parikh et al., 2024; Tasiou et al., 2025; Wariani et al., 2020; Helling and McKinlay, 2005; Beda et al., 2007; Huh, 2013; Willson et al., 2014; Chue et al., 2016; Dukes et al., 2013; Johannigman et al., 2015; Andersson et al., 2003; Brändström et al., 2017; Lim et al., 2020; Bichsel et al., 2022; Donovan et al., 2008).

**Table 1:** Decision-relevant evidence for aeromedical transfer after severe TBI, cranial surgery, or pneumocephalus.

Study	Design/population	Main finding	Interpretation/limitations
Goodman et al. (2010)	Narrative review of TBI and aeromedical evacuation	Hypobaric exposure could act as an additional physiological/inflammatory insult; prioritizes stabilization and secondary-injury prevention.	Mechanistic and expert-opinion basis; no validated clearance interval.
Fang et al. (2010)	Operational review of intercontinental military TBI evacuation	Safe movement depends on selection, mission preparation, trained critical-care teams, equipment, and communication.	Military system; limited direct civilian generalizability.
Dukes et al. (2013)	Retrospective CCATT cohort; 63 severe isolated TBI casualties	53% experienced at least one documented secondary insult; hyperthermia was most common and hypoxia was uncommon.	Retrospective charting; older military practice.
Johannigman et al. 2015	Observational series; 11 TBI patients with continuous ICP/ABP monitoring	ICP commonly changed during movement/flight; most elevations were transient and 5 patients never exceeded 20 mm Hg.	Small sample; no control group.
Boyd et al. 2017	Retrospective severe-TBI CCATT series	Ventilation, vasopressors, and management of ICP, diabetes insipidus, and seizures were frequently required; no in-flight deaths were reported.	Retrospective and military; short-term outcomes.
Maddry et al. (2020a)	Retrospective CCATT cohort; 435 moderate/severe TBI	31% used cabin-altitude restriction; 19% had preflight PaO_2_ <80 mm Hg and 3% had in-flight SpO_2_ <93%. No clear mortality, discharge, or length-of-stay advantage from restriction was demonstrated.	Confounding by indication; not a trial of cabin altitude.
Maddry et al. (2020b)	Retrospective 7-year multicentre cohort; 438 combat casualties	Evacuation ≥3 days after injury was associated with lower adjusted mortality than evacuation within 1 day.	Association is vulnerable to survivor and selection bias; not a causal safe-time threshold.
Davis et al. (2022)	Retrospective en-route cohort; 404 TBI casualties	In-flight SBP <100 mm Hg occurred in 13.9%; among 120 with ICP monitoring, CPP <60 mm Hg occurred in 30.8%.	Episodic documentation may underestimate events.
Ng et al. (2023)	Retrospective evacuation-strategy study; overlapping 438-casualty cohort	Earlier evacuation was associated with more hypotension/interventions in some subgroups; mortality did not differ significantly across timing groups.	Population overlaps with Maddry et al.; results must not be pooled as independent.
Powell and Cramb (2025)	Retrospective civilian aeromedical trauma cohort; 2927 transports	Median time to first hypotension was 11.5 min; risk plateaued at about 50 min. Isolated TBI had more in-flight hypotension than other trauma (39.5% vs 9.2%).	Not restricted to postoperative or decompressive-craniectomy patients.
Andersson et al. (2003)	Computer model of intracranial air and cabin pressure	Predicted substantial gas expansion and ICP rise depending on starting air volume and intracranial compliance.	Model-derived; cannot define a clinical volume cutoff.
Brändström et al. (2017)	Retrospective 119 post-craniotomy patients plus simulation; 34 had pneumocephalus	Model suggested risk at larger volumes; authors emphasized recent CT and consideration of sea-level cabin pressure.	No observed in-flight outcome validation; proposed values are model-dependent.
Lim et al. (2020)	Bench model of pneumocephalus under simulated cabin ascent	Proposed combinations of air volume, initial ICP, and ascent rate associated with pressure rise.	Experimental proposal, not a validated clinical threshold.
Bichsel et al. (2022)	Systematic review; 13 included reports plus 5 from citation searching	No reported decompensation clearly attributable to pre-existing intracranial air during flight; persistent extracranial–intracranial fistula appeared more important than volume alone.	Sparse heterogeneous evidence and likely publication bias.
Donovan et al. (2008)	Case series; 21 patients with 0.6–42.7 mL intracranial air	No neurological decline during aeromedical evacuation; 3 monitored patients had no sustained ICP elevation.	Small selected series with stable patients; does not prove safety.
Amato-Watkins et al. (2013)	UK consultant-neurosurgeon survey	Advice ranged from <2 to >8 weeks; pneumocephalus was the dominant concern.	Opinion and practice variation, not outcome evidence.
Mampre et al., (2026)	US neurosurgeon case-scenario survey	Mean suggested clearance after craniectomy with bone flap off was 2.7 weeks, but variability was wide and decisions strongly depended on pneumocephalus volume.	Survey of opinion; no outcome validation.
Tasiou et al. (2025)	Multidisciplinary framework/review for acutely unwell neurosurgical patients	Emphasizes stabilization, platform choice, equipment, trained escorts, communication, and checklists.	Framework derived from limited heterogeneous evidence.

Abbreviations: ABP, arterial blood pressure; CCATT, Critical Care Air Transport Team; CPP, cerebral perfusion pressure; ICP, intracranial pressure; SBP, systolic blood pressure; TBI, traumatic brain injury.

The evidence base comprised retrospective military and civilian cohorts, small physiological series, surveys, case reports/series, reviews, and computer/bench models. No randomized trial or prospective validation study defined a postoperative flight interval, a quantitative intracranial-air threshold, or an aeromedical clearance score. Several reports from the US combat casualty system used overlapping 2007–2014 populations and were therefore interpreted as complementary analyses rather than independent samples (Maddry et al., 2020b; Davis et al., 2022; Ng et al., 2023; Powell and Cramb, 2025).

### 5.2 Timing and clinical outcomes

Timing data do not establish a universal safe day to transport patient after DC/cranial surgery. In 438 combat casualties, evacuation at ≥3 days was associated with lower adjusted mortality than evacuation within 1 day (Maddry et al., 2020b), while a related analysis found more hypotension and interventions in selected early-evacuation subgroups without a significant mortality difference (Ng et al., 2023). These observational associations are vulnerable to survivor bias, injury-severity differences, evolving resuscitation, and confounding by indication. Surveys likewise show practice rather than evidence based safety decisions: A UK survey conducted in 2013 revealed that 5 out of 66 neurosurgeons were against air travel after neurosurgery, with the remaining 61 recommending an air travel restriction period ranging from 2 to 8 weeks (Amato-Watkins et al., 2013). In a recently published US survey, recommended flight-clearance times by neurosurgeons varied by procedure: tumor craniotomy, [mean (SD)] 2.4 (2.2) wk; subdural haematoma craniotomy, 3.8 (3.5) wk; uncomplicated transparanasal approach, 4.4 (3.7) wk; transparanasal approach with cerebrospinal fluid leak repair, 6.0 (4.3) wk; craniectomy with bone flap off, 2.7 (3.0) wk; and ventriculoperitoneal shunt, 2.0 (1.6) wk. Authors concluded that variability of responses highlights the need for further research as well as surgeon education on altitude physiology and aeromedical risk factors (Mampre et al., 2026).

### 5.3 Secondary insults during transport

Secondary physiological insults were common enough to justify ICU-level planning. Dukes et al. reported at least one insult in 53% of 63 severe isolated TBI transports, most commonly hyperthermia (Dukes et al., 2013). In a small continuously monitored cohort, ICP varied during movement/flight, although many changes were transient (Johannigman et al., 2015). Among 404 TBI casualties, in-flight SBP <100 mm Hg occurred in 13.9%; CPP <60 mm Hg occurred in 30.8% of the 120 patients with ICP monitoring (Davis et al., 2022). In a recent paper examining the association between flight duration and the risk of in-flight deterioration in aeromedical trauma patients in a 2927-patient civilian trauma cohort, hypotension tended to occur early (median 11.5 min), and isolated TBI patients had a higher event rate than other trauma patients (39.5% vs 9.2%) (Powell and Cramb, 2025). These findings support preflight stabilization, continuous monitoring, early vigilance, and immediate treatment capability; they do not prove that flight itself caused each event.

### 5.4 Altitude, oxygenation, and cabin strategy

In a study to examine the effects of cabin altitude restriction on 435 moderate to severe TBI patients transported out of theater via Critical Care Air Transport Teams, 31% received cabin-altitude restriction, 19% had a preflight PaO_2_ <80 mm Hg, and 3% had an in-flight SpO_2_ <93% (Maddry et al., 2020a). Cabin restriction was not associated with better mortality, discharge disposition, or length of stay, but the comparison was non-randomized and confounded by selection. Operational reports consistently emphasize adequate oxygen reserve, ventilation, haemodynamics, equipment, and team competence (Goodman et al., 2010; Fang et al., 2010; Boyd et al., 2017; Parikh et al., 2024; Tasiou et al., 2025; Wariani et al., 2020).

### 5.5 Pneumocephalus and postoperative cranial surgery

Boyle's law predicts expansion of enclosed gas as ambient pressure falls. Computer and bench models show that the ICP effect depends on initial air volume, baseline ICP/compliance, ascent rate, and simulated cabin altitude (Andersson et al., 2003; Brändström et al., 2017; Lim et al., 2020). Proposed values such as 11, 20, or 30 mL are therefore model-dependent and should not be presented as validated clinical gates. In contrast, 21 selected patients with 0.6–42.7 mL intracranial air had no neurological decline during aeromedical evacuation (Donovan et al., 2008), and a small series after malignant-glioma surgery reported only minor cerebral-metabolic changes during transport (Lindvall et al., 2008). A systematic review found no clearly documented deterioration caused solely by pre-existing pneumocephalus during flight (Bichsel et al., 2022). Case reports have described pneumocephalus or neurological symptoms after commercial air travel, particularly when skull-base/sinus defects or possible extracranial–intracranial fistulae were present (Helling and McKinlay, 2005; Beda et al., 2007; Huh, 2013; Willson et al., 2014). The clinically important questions are thus not volume alone, but mass effect/tension physiology, evolution on imaging, baseline intracranial compliance, and a persistent route for air entry.

### 5.6 Current guidance/guidelines on aeromedical transfers after DC for TBI

No worldwide protocol specifically validates transfer after DC. Aerospace Medical Association guidance suggests that 10–14 days of observation with assessment for intracranial air and neurological stability may be appropriate after intracranial surgery (Aerospace Medical Association Medical Guidelines Task Force, 2014); the wording is advisory and primarily addresses passenger travel. British Thoracic Society recommendations guidance advises commercial passengers not to fly until 7 days after full radiographic resolution of pneumothorax (Coker et al., 2022), and provides practical advice for healthcare professionals in primary and secondary care managing passengers with pre-existing respiratory conditions planning commercial air travel, including those recovering from an acute event/exacerbation. Severe-TBI and safe-transfer guidelines focus on preventing hypoxaemia, hypotension, deranged ventilation, fever, seizure, and loss of cerebral perfusion, and on trained escorts, monitoring, equipment, and agreed clinical responsibility (Carney et al., 2017; Hawryluk et al., 2019; Nathanson et al., 2020). Table 2 compares current guidance and practice relevant to aeromedical transfer and its limitations. Updated, pragmatic advices regarding TBI patients, and more generally neurosurgical patients, requiring air transfer is still missing, and time has come for clinical statements by the appropriate stakeholders and scientific societies.

**Table 2:** Comparison of current guidance and practice relevant to aeromedical transfer.

Source	Scope	Relevant position	Important caveat
Aerospace Medical Association, 2014 (Aerospace Medical Association Medical Guidelines Task Force, 2014	Commercial passenger travel after intracranial surgery	Suggests that 10–14 days of observation with assessment for intracranial air and neurological stability may be appropriate.	Advisory language; not specific to ventilated air-ambulance transfer and not a validated minimum.
UK neurosurgeon survey, 2013 (Amato-Watkins et al., 2013) and airline survey, 2009 (Seth et al., 2009)	Post-craniotomy commercial flying	Shows wide practice and airline-policy variation; intracranial air is the main concern.	Describes opinion/policy rather than clinical outcomes.
US neurosurgeon survey, 2026 (Mampre et al., 2026)	Procedure-specific return to commercial flight	Mean advice varies by procedure and pneumocephalus volume; craniectomy responses remain highly variable.	Recent but still expert opinion.
BTS Clinical Statement, 2022 (Coker et al., 2022)	Commercial passenger travel after pneumothorax	Do not fly until 7 days after full radiographic resolution.	Passenger guidance; urgent ICU air ambulance with a functioning drain requires specialist case-by-case planning.
Brain Trauma Foundation, 2017 (Carney et al., 2017); SIBICC, 2019 (Hawryluk et al., 2019)	Severe-TBI physiological management	Avoid hypotension and hypoxaemia; maintain individualized ICP/CPP, oxygenation, ventilation, temperature, and seizure targets.	TBI-care standards, not flight-clearance rules.
Association of Anaesthetists/NACCS, 2020 (Nathanson et al., 2020)	Interhospital transfer of brain-injured patients	Resuscitate and stabilize before transfer; use trained escorts, appropriate monitoring/equipment, and agreed clinical responsibility.	Mixed evidence and consensus; principally ground/interhospital transfer.
Published CCATT experience (Fang et al., 2010; Dukes et al., 2013; Johannigman et al., 2015; Boyd et al., 2017; Maddry et al., 2020a, 2020b; Davis et al., 2022; Ng et al., 2023)	Long-range critical-care military evacuation	Demonstrates feasibility with ICU-capable teams, cabin-altitude restriction when selected, and active prevention/treatment of secondary insults.	Specialized military system and overlapping retrospective cohorts.
India: Immigration and Foreigners Rules, 2025 (Government of India and Ministry of Home Affairs, 1939; Government of India and Ministry of Home Affairs, 2025)	Administrative duties for foreign/OCI patients	Hospitals record arrival/departure details, transmit Form III electronically, and retain records as prescribed.	Administrative requirement; local legal/FRRO advice should be checked for the specific case.

Abbreviations: UK, United Kingdom; US, United States; BTS, British Thoracic Society; SIBICC, Seattle International Severe Traumatic Brain Injury Consensus Conference; NACCS, the Neuro Anaesthesia and Critical Care Society; CCATT, Critical Care Air Transport Team; TBI, Traumatic Brain Injury; OCI, Overseas Citizenship of India; ICP, intracranial pressure; CPP, cerebral perfusion pressure; ICU, intensive care unit; FRRO, Foreigners Regional Registration Office.

## 6 Discussion

Global air travel involves billions of passengers annually, including those requiring medical repatriation. In patients with recent neurosurgical interventions, aeromedical transfer poses significant physiological risks due to hypobaric conditions in flight, along with complex logistical, financial, and diplomatic challenges. Adequate travel insurance is critical, as the cost of critical care and international aeromedical repatriation can be substantial. In the present case, treatment in a public hospital in India incurred no direct charges to the patient, though similar care in private or international settings may be prohibitively expensive. Comparable treatment in a private setting was estimated to cost approximately INR 2.5 million or higher ( about € 25000 or higher) for a three -week hospital stay, including surgery.

Current literature cannot support a universal waiting interval or intracranial-air volume cutoff before air transport of a patient with severe TBI and DC. A status-based decision is more defensible than a calendar-based rule. Present case demonstrates feasibility under one carefully prepared set of circumstances, not a universal clearance rule. The patient's readiness depended on stable neurological trajectory, reassuring repeat CT, radiographic resolution of pneumothorax, stable room-air gas exchange, secure tracheostomy, a critical-care aircraft and escort, and formal receiving-centre acceptance. This case highlights a practical but poorly standardised problem: deciding fitness to fly after decompressive craniectomy for severe TBI. Patients after craniectomy or recent neurosurgical intervention may require interfacility or international transfer, but the decision should not be based only on the number of postoperative days (Wariani et al., 2020; Walcott et al., 2011). It should be based on structured physiological and radiological assessments as detailed below (Wallace and Ridley, 1999; Sethi and Subramanian, 2014; Kulshrestha and Singh, 2016).

Cruising at an altitude of 35,000 feet poses a significant clinical risk to patients with pneumocephalus because commercial aircraft and air ambulances typically maintain a cabin pressure equivalent to an altitude of 6000–8000 feet rather than sea level. According to Boyle's Law, the volume of a trapped gas is inversely proportional to its surrounding pressure; therefore, this reduction in ambient pressure causes a predictable expansion of intracranial or intrathoracic air by approximately 25–30% during flight (Andersson et al., 2003; Brändström et al., 2017; Lim et al., 2020; Bichsel et al., 2022). Under normal physiological conditions, the body typically reabsorbs intracranial air within two to five days; however, air may persist longer depending on the nature of the injury or surgery. For patients who have undergone DC, the presence of postoperative residual air (pneumocephalus) is high risk situation during air travel. In a patient after cranial surgery, this becomes clinically important if residual pneumocephalus, CSF rhinorrhea, or uncontrolled intracranial pressure is present. Expansion of intracranial air can increase intracranial pressure and tension pneumocephalus (Andersson et al., 2003; Lim et al., 2020; Bichsel et al., 2022). Visible “mushrooming” or bulge of the scalp flap after DC may be an early warning sign of pressure changes beneath the flap. Clinicians should perform a careful, case by case basis evaluation of initial intracranial injury, the presence of concomitant injuries or comorbidities, current clinical scenario, especially assessing for any intracranial air volume or possible CSF rhinorrhea. The risk of increase in ICP would in fact depend on initial air volume, the starting ICP value, and the rate of increase in cabin altitude (Brändström et al., 2017). Performing a postoperative head CT and estimate intracranial air volumes for post-intracranial surgery patients is therefore imperative for verification of the presence or absence of intracranial air after craniotomy or the presence of extracranial to intracranial fistulous process for patients planned for long distance air transport. Experimental and modelling studies suggest that larger intracranial air volumes (30 mL or more) may produce clinically important pressure increases during cabin-altitude exposure; however, no universally validated volume threshold currently defines fitness to fly (Andersson et al., 2003; Brändström et al., 2017). In our patient, transfer was considered only after CT showed no residual haematoma, hydrocephalus, significant mass effect, or pneumocephalus.

A second issue is maintenance of cerebral and systemic homeostasis during transfer. Hypobaric hypoxia, even in a pressurized cabin, can reduce oxygen reserve in a recently injured brain. Oxygenation should be monitored continuously, ventilation should aim for normocapnia, avoiding both hypercapnia-related intracranial pressure elevation. End-tidal carbon dioxide may not always accurately reflect arterial carbon dioxide during altitude exposure or transport ventilation, so recent arterial blood gas analysis is useful before departure (Wallace and Ridley, 1999; Sethi and Subramanian, 2014; Kulshrestha and Singh, 2016). Positioning with head elevation and neutral neck alignment should be maintained as far as practicable during ground and air transfer. Airway-device security, tracheostomy cuff-pressure surveillance, aspiration prevention, and availability of rescue airway equipment are essential during altitude exposure.

Safe repatriation requires clear communication between treating hospital, aeromedical provider, receiving hospital, insurer, embassy or consulate, and family (Wallace and Ridley, 1999; Sethi and Subramanian, 2014; Kulshrestha and Singh, 2016). Relevant imaging, operative notes, ICU records, medication charts, microbiology reports, and transfer instructions should accompany the patient. For patients with stored autologous bone flaps, documentation of ownership, sterility, packaging, and cold chain is ensured. A structured handover to the receiving team is critical to ensure continuity of care and ongoing neuroprotective management (Helling and McKinlay, 2005; Israëls et al., 2018).

**Practical fitness to fly pathway**: The proposed and unvalidated structured decision pathway for intercontinental aeromedical transfer after decompressive craniectomy is presented in Table 3, Table 4.

**Table 3:** Objective algorithm for intercontinental aeromedical transfer after decompressive craniectomy for traumatic brain injury. All decision nodes are objective PASS/FAIL gates requiring recorded values. Any FAIL stops transfer until corrected and reassessed.

TIME FROM LAST CRANIAL OPERATION	INTRACRANIAL AIR RULE	CHEST AIR RULE
<7 days: DEFER 7-21 days: CONDITIONAL only if all gates PASS >21 days: PROCEED only if all gates PASS	>30 mL, tension pneumocephalus, or air-associated mass effect: DEFER 5-30 mL with no mass effect: CONDITIONAL with written low-cabin-altitude/sea-level pressurization plan <5 mL or none: PASS	Undrained pneumothorax or persistent air leak: DEFER Chest drain present + no bubbling for≥24 h + residual pneumothorax <10%: CONDITIONAL No pneumothorax: PASS
Record operation date/time and planned take-off date/time.	Measure air on PACS volumetry; if unavailable, use ABC/2 for the largest air pocket.	Chest X-ray/CT within 24 h before departure if thoracic injury or chest tube is present.

Gate	Objective domain	PASS requires ALL listed values	If PASS	If FAIL
**A**	Neurological deterioration screen	Last 24 h: no fall in GCS motor score≥1; no new pupillary asymmetry or loss of reactivity; no new focal motor deficit; no clinical seizure/status epilepticus.	**Go to B**	DEFER: urgent clinical examination; CT head; EEG if seizure suspected; treat cause and reassess.
**B**	Non-contrast CT head within 24 h	No new/enlarging haematoma; midline shift not increased by≥2 mm vs prior CT; basal cisterns patent; no radiological herniation; no untreated hydrocephalus; intracranial air rule PASS or CONDITIONAL.	**Go to C**	DEFER: treat lesion/air/hydrocephalus; repeat CT before transfer.
**C**	Respiratory + arterial blood gas (ABG)	SpO2≥94% for≥6 h on planned support; ABG within 6 h: pH 7.35-7.45, PaO2 80-100 mm Hg or neuromonitor-guided target, PaCO2 35-45 mm Hg; FiO2≤0.60; PEEP≤10 cm H2O; EtCO2 available.	**Go to D**	DEFER: correct oxygenation/ventilation; repeat ABG.
**D**	Haemodynamics	SBP≥110 mm Hg; no vasopressor initiation/escalation in last 24 h; HR 60-120/min; core temperature 36.0-37.9 C; urine output≥0.5 mL kg-1 h-1.	**Go to E**	DEFER: resuscitate; treat arrhythmia/fever/shock; reassess after thresholds are stable for≥6 h.
**E**	Laboratory thresholds	Hb≥7 g/dL; Na 135-145 mEq/L; K 3.5-5.5 mEq/L; glucose 100-180 mg/dL; no active bleeding or transfusion/intervention in last 6 h.	**Go to F**	DEFER: correct abnormality; repeat laboratory tests.
**F**	Airway/lines/tubes	Endotracheal tube/tracheostomy secured; cuff pressure 20-30 cm H2O; suction available; two functioning IV lines or central line; all drains/catheters secured; spare airway/tracheostomy kit present.	**Go to G**	DEFER: replace/secure device and document function.
**G**	Documents + receiving hospital	Transfer summary complete; current neurological examination, ABG, ventilator settings, infusion rates, culture/antibiotic plan documented; CT images/reports sent; if preserved bone flap is transferred, sterile packaging, appropriate labelling, and written storage instruction at −20°C documented; receiving ICU bed plus named neurosurgeon/intensivist acceptance documented.	**Go to H**	DEFER: complete records transfer and written acceptance.
**H**	Cabin + transport platform	Air ambulance ventilator supports current settings; oxygen reserve≥2 times calculated requirement; monitor includes ECG, SpO2, non-invasive/invasive BP, EtCO2 and temperature; cabin-altitude plan documented.	**FIT TO FLY**	DEFER: upgrade aircraft/ambulance/resources or revise route.

FINAL DECISION: FIT TO FLY = Gate A through H PASS + any CONDITIONAL air/cabin item has a written mitigation plan + mandatory signatures by the treating neurosurgeon, intensivist/anaesthesiologist, and flight physician. Any single FAIL = DO NOT FLY until the objective threshold is met and documented.

**Abbreviation:** ABG, arterial blood gas; BP, blood pressure; CT, computed tomography; ECG, electrocardiography; EtCO2, end-tidal carbon dioxide; FiO2, fraction of inspired oxygen; GCS, Glasgow Coma Scale; Hb, haemoglobin; HR, heart rate; ICU, intensive care unit; IV, intravenous; PACS, picture archiving and communication system; PaCO2, arterial carbon dioxide tension; PaO2, arterial oxygen tension; PEEP, positive end-expiratory pressure; SBP, systolic blood pressure; SpO2, peripheral oxygen saturation. PASS indicates that the minimum objective threshold has been met for proceeding to the next gate; CONDITIONAL indicates that transfer may proceed only with a documented mitigation plan; DEFER/FAIL indicates that transfer should be delayed until the abnormality is corrected and reassessed.

**Evidence base:** postoperative timing and clearance (Amato-Watkins et al., 2013; Mampre et al., 2026; Seth et al., 2009; Maddry et al., 2020b; Aerospace Medical Association Medical Guidelines Task Force, 2014); intracranial air and cabin-pressure evidence (Andersson et al., 2003; Brändström et al., 2017; Lim et al., 2020; Bichsel et al., 2022; Donovan et al., 2008; Lindvall et al., 2008; Helling and McKinlay, 2005; Beda et al., 2007; Huh, 2013; Willson et al., 2014; Chue et al., 2016; Aerospace Medical Association Medical Guidelines Task Force, 2014); thoracic-air guidance (Coker et al., 2022); and severe-TBI physiology, monitoring, and transfer practice (Goodman et al., 2010; Fang et al., 2010; Dukes et al., 2013; Johannigman et al., 2015; Boyd et al., 2017; Maddry et al., 2020a, 2020b; Davis et al., 2022; Ng et al., 2023; Powell and Cramb, 2025; Parikh et al., 2024; Tasiou et al., 2025; Wariani et al., 2020; Carney et al., 2017; Hawryluk et al., 2019; Nathanson et al., 2020; Walcott et al., 2011; Wallace and Ridley, 1999; Sethi and Subramanian, 2014; Kulshrestha and Singh, 2016; Israëls et al., 2018; Mazzeo et al., 2026). The numerical thresholds are evidence-informed operational criteria and have not been prospectively validated.

**Table 4:** **Evidence-informed defer/conditional/ready pathway for fixed-wing transfer after severe TBI and decompressive craniectomy. Evidence base:** aeromedical, cranial-surgery, and pneumocephalus evidence (Goodman et al., 2010; Fang et al., 2010; Dukes et al., 2013; Johannigman et al., 2015; Andersson et al., 2003; Brändström et al., 2017; Lim et al., 2020; Bichsel et al., 2022; Donovan et al., 2008; Lindvall et al., 2008; Amato-Watkins et al., 2013; Mampre et al., 2026; Seth et al., 2009; Boyd et al., 2017; Maddry et al., 2020a, 2020b; Davis et al., 2022; Ng et al., 2023; Powell and Cramb, 2025; Parikh et al., 2024; Tasiou et al., 2025; Wariani et al., 2020; Helling and McKinlay, 2005; Beda et al., 2007; Huh, 2013; Willson et al., 2014; Chue et al., 2016; Walcott et al., 2011; Wallace and Ridley, 1999; Sethi and Subramanian, 2014; Kulshrestha and Singh, 2016; Israëls et al., 2018; Mazzeo et al., 2026); professional guidance (Aerospace Medical Association Medical Guidelines Task Force, 2014; Coker et al., 2022; Carney et al., 2017; Hawryluk et al., 2019; Nathanson et al., 2020); and cross-border legal sources (Government of India and Ministry of Home Affairs, 1939; Government of India and Ministry of Home Affairs, 2025).

Domain	DEFER	CONDITIONAL	READY
1. Clinical purpose and urgency	No receiving indication/acceptance; transfer risk exceeds expected benefit.	Time-sensitive transfer despite recent surgery, with documented benefit–risk decision.	Clinical goal, receiving bed, responsible consultants, and contingency destinations are agreed.
2. Neurological trajectory	Deteriorating GCS/motor response, new pupillary change, uncontrolled seizure, herniation, or uncontrolled ICP/CPP.	Stable but deeply impaired examination, ongoing sedation, or ICP therapy that can be reproduced in flight.	Stable serial examination; no new seizure; current neuroprotective targets and rescue plan documented.
3. Cranial imaging and intracranial air	New/enlarging haemorrhage, mass effect, hydrocephalus, tension pneumocephalus, or active CSF/air fistula.	Small, stable intracranial air without mass effect; transfer only after neurosurgical review and a cabin-pressure/oxygen plan.	Recent CT reviewed; no concerning air, mass effect, new bleed, or suspected fistula. Do not use a single air-volume cutoff as a validated gate.
4. Thorax and trapped gas	Untreated pneumothorax, enlarging residual air, or persistent uncontrolled air leak.	Chest drain in situ with confirmed function and no uncontrolled leak; specialist plan and drainage equipment required.	Lung expanded and clinically stable; recent imaging/ultrasound reviewed. Apply commercial-passenger waiting rules cautiously to ICU air ambulance.
5. Airway and ventilation	Unsecured/unstable airway, frequent plugging, unmanageable secretions, or ventilatory support unavailable on the platform.	Tracheostomy or invasive ventilation is stable but requires active suction, cuff surveillance, and backup airway plan.	Airway position/fixation verified; spare tube, suction, bag-mask device, capnography, ventilator, and backup oxygen checked.
6. Oxygenation and CO_2_	Refractory hypoxaemia, worsening gas exchange, or CO_2_ outside the prescribed neurocritical target despite support.	Stable higher oxygen/PEEP requirement with adequate reserve and cabin-altitude mitigation.	Stable trend and recent ABG when indicated; target oxygenation and PaCO_2_/EtCO_2_ range written for the escort team.
7. Haemodynamics and metabolism	Active bleeding, escalating vasopressor requirement, recurrent hypotension, severe temperature/electrolyte/glucose disturbance.	Low stable vasopressor dose or correctable abnormality with adequate in-flight reserve.	Individualized SBP/MAP/CPP and temperature targets documented; adequate fluids, blood products/vasopressors, and point-of-care capability available as needed.
8. Seizure, sedation, and comfort	Ongoing status epilepticus, uncontrolled agitation, or no safe sedation/analgesia plan.	Recent controlled seizure or deep sedation; rescue drugs and dosing schedule required.	Scheduled antiseizure medicines given; sedation, analgesia, antiemetic, and rescue plans checked.
9. Lines, drains, nutrition, and pressure care	Essential access/drain insecure or likely to run out; unresolved device problem.	Complex devices or long mission with higher pressure-injury/aspiration risk.	Two reliable access routes when appropriate; lines labelled and secured; feed/aspiration plan, pressure care, urine output, and DVT prevention addressed.
10. Aircraft and cabin plan	Aircraft cannot meet oxygen, power, space, monitoring, weight, or cabin-altitude needs.	Cabin-altitude restriction/sea-level cabin requested; fuel/range implications accepted and documented.	Aircraft performance and cabin profile confirmed by operator; ascent/descent risks and diversion plan briefed.
11. Escort team and equipment	No clinician competent in neurocritical care/airway/ventilation or no functioning backup equipment.	Capability gap can be closed by an additional escort or equipment change.	Role allocation, preflight checklist, medications, batteries, oxygen reserve, PPE, and emergency rehearsals complete.
12. Handover, documents, and legal steps	Receiving team has not accepted; essential records/consent/identity/immigration steps incomplete.	Non-critical document pending with named owner and deadline before departure.	Imaging, operative/ICU summary, medication chart, consent, fit-to-fly plan, contact numbers, passport/visa and required reporting are complete; bone-flap cold chain follows the originating and receiving institutions' protocols.

**Use:** Any unmitigated DEFER item postpones transfer. CONDITIONAL items require named specialist review and a written mitigation plan. This pathway is not a validated score; clinical urgency may change the benefit–risk balance.

Abbreviations: GCS, Glasgow Coma Scale; ICP, intracranial pressure; CPP, cerebral perfusion pressure; CSF, cerebrospinal fluid; PEEP, positive end expiratory pressure; CT, computed tomography; ICU, intensive care unit; ABG, arterial blood gases; SBP/MAP/CPP, systolic blood pressure, mean arterial pressure, cerebral perfusion pressure, DVT, deep venous thrombosis; PPE, personal protective equipment.

Air transfers should be deferred in presence of unmitigated hazard such as neurological deterioration, tension pneumocephalus, active air fistula, untreated pneumothorax, refractory hypoxaemia, recurrent hypotension, uncontrolled seizure, or an inadequate aircraft/team. Air transfer may be conditional at times and justified only after specialist review and a written mitigation plan—for example, small stable intracranial air, a functioning chest drain, a recent but time-sensitive postoperative transfer, a stable vasopressor requirement, or the need for cabin-altitude restriction. Air transfer is permitted when every domain is stable and the team, platform, receiving centre, documentation, and contingency plan are complete (Table 4).

A recent CT is appropriate when the result could change clearance, cabin profile, or neurosurgical management. The scan should be reviewed for new haemorrhage, mass effect, hydrocephalus, tension pneumocephalus, and evidence of a skull-base or wound fistula. A small stable collection without mass effect may be a conditional rather than absolute contraindication in a fully supported air ambulance, but the decision must account for the anticipated cabin altitude and intracranial reserve. When risk remains material, a lower cabin altitude or sea-level cabin request may reduce gas expansion and hypobaric hypoxia, at the cost of range, fuel, and mission complexity.

Untreated pneumothorax or an uncontrolled air leak should defer flight. Where urgent medical transport is essential, a functioning chest drain, confirmation of lung expansion, drain accessibility, and an in-flight plan for recurrence are critical.

Safe transfer requires a named clinical lead at the referring and receiving centres, formal acceptance, a shared physiological plan, imaging and operative records, medication and device schedules, direct contact numbers, and a diversion/escalation plan. Insurer and aeromedical-provider coordination should occur in parallel with clinical review and should not determine fitness to fly. Bone-flap packaging, temperature, chain of custody, labelling, and acceptance should follow the written protocols of both institutions; no universal −20°C transport rule was identified.

A pressurized fixed-wing air ambulance configured for critical-care transport is preferred for long-distance transfer, particularly when cabin-altitude limitation is clinically important. Transport ventilators should support volume- or pressure-controlled modes with positive end expiratory (PEEP) and alarms, and settings should approximate ICU parameters, with provision for arterial blood gas–guided adjustments. Continuous monitoring—including ECG, blood pressure (preferably invasive), oxygen saturation, end-tidal CO_2_, and temperature—is essential; and when available, intracranial pressure monitoring should be continued with predefined drainage protocols.

Comprehensive preparation includes ensuring availability of essential medications (sedatives, analgesics, vasopressors, anticonvulsants, osmotherapy agents, and emergency drugs), along with backup airway equipment, including spare tracheostomy tubes and chest tubes for possible pneumothorax drainage. The escort team should consist of personnel experienced in neurocritical care and airway management, with pre-flight and inflight clear communication established with the receiving center.

During flight, maintenance of neuroprotection strategies and of systemic homeostasis is paramount. Adequate oxygenation (SpO_2_ ⩾ 94%, individualized when appropriate) should be maintained by adjusting inspiratory fraction of oxygen and PEEP to counter hypobaric hypoxia. Ventilation should target normocapnia to avoid ICP fluctuations, recognizing that end-tidal CO_2_ may underestimate PaCO_2_ at high altitudes. Hemodynamic stability must be ensured to maintain cerebral perfusion pressure. Special attention is required for tracheostomy care, including secure fixation, maintenance of patency with regular suctioning, and careful cuff pressure monitoring due to gas expansion at high altitudes. Preparedness for airway emergencies, including accidental decannulation, is essential. Additional precautions include minimizing ventilator disconnections, preventing aspiration, and ensuring all lines, drains, and devices are securely fixed.

Admission of a foreign national requires compliance with foreigner-registration requirements under the Registration of Foreigners framework, including reporting within 24 h of admission in the hospital. Hospitals should document passport, visa, local address, attendant details, admission particulars, and intended stay, with submission of the prescribed forms. This becomes relevant when repatriation is planned after days or weeks, as immigration status, visa validity, discharge documentation, medical fitness, and aeromedical clearance must be coordinated before safe transfer (Government of India and Ministry of Home Affairs, 1939; Government of India and Ministry of Home Affairs, 2025). Each hospital should have structured protocols to manage international emergency patients according to national regulations.

An Indian patient sustaining traumatic brain injury in Europe, for example, would require coordination between the treating hospital, local authorities, Indian Embassy/Consulate, insurer, aeromedical provider, airline medical services, family, and receiving hospital in India. Repatriation should ultimately be guided by neurological stability and transport safety.

We propose that transcontinental transfers should not be solely based on elapsed time from surgery; it should proceed only after sequential PASS/FAIL and/or Defer/Conditional/Ready assessments (Table 3, Table 4). Any failed gate should defer transfer until the abnormality is corrected and reassessed, and any conditional air or cabin-pressure concern should have a written mitigation plan before departure. The pathway is evidence-informed and should be audited prospectively before being called a protocol.

### 6.1 Limitations

The clinical report describes one patient and lacks continuous high-resolution in-flight physiological data. Included evidence from systematic literature review is heterogeneous, largely retrospective and vulnerable to selection, survivor, reporting, and publication bias. Several cohorts overlap, no meta-analysis was appropriate, formal certainty grading was not performed, and the proposed pathway has not been prospectively validated.

## 7 Conclusions

Intercontinental air transfer after decompressive craniectomy in severe TBI patients poses specific challenges related to the transport of a patient under hypobaric conditions. A structured multidisciplinary fitness-to-fly pathway may reduce preventable risk. Current evidence supports individualized assessment of neurological trajectory, recent cranial and thoracic imaging, intracranial or pleural air and fistula risk, oxygenation/ventilation reserve, haemodynamics, seizure control, airway and devices, aircraft/cabin capability, escort competence, receiving-centre readiness, and cross-border documentation.

A consensus for healthcare professionals managing patients or passengers with recent neurotrauma or neurosurgical conditions planning commercial air travel or aeromedical transport is needed consistent with existing guidance for respiratory diseases.

## Patient consent

Written informed consent for publication was obtained from the patient's legally authorised representative. All clinical information was anonymised. Institutional ethics-review requirements for publication of an individual case were followed.

## Author's contribution

DG: Conceptualization; Data curation; Supervision; Conducted PRISMA systematic review Validation; Visualization; Writing – review, literature screening, verification, and risk-of-bias appraisal & editing; RK: data curation, writing- original draft preparation; RS: visualization, investigation; KP: visualization, investigation; VG: Writing - review & editing; AK: visualization, investigation; BG: Data curation; investigation; KF: supervision; ATM: conceptualization; writing-reviewing, literature screening, verification, and risk-of-bias appraisal and editing.

## Ethics

The patient's identity has been anonymised and not disclosed.

## Funding

No funding was received for this work.

## Declaration of competing interest

The authors declare that they have no known competing financial interests or personal relationships that could have appeared to influence the work reported in this paper.
