# Supplementary material for: Intercontinental aeromedical transfers after decompressive craniectomy for severe traumatic brain injury: Systematic review and evidence-informed fitness-to-fly pathway
Source: Brain Spine. 2026 Aug 9;6:106278. doi: 10.1016/j.bas.2026.106278 (PMC13486883; doi:10.1016/j.bas.2026.106278)
Supplement: Supplementary file 1 [file mmc1.docx]

**Supplementary Table 1. Search strategies used for identification of studies**

| **Database** | **Date searched** | **Search strategy** | **Records retrieved** |
| --- | --- | --- | --- |
| **PubMed (MEDLINE)** | 24 July 2026 | ( ("Brain Injuries, Traumatic"[Mesh] OR "traumatic brain injury" OR TBI OR "head injury") OR ("Neurosurgical Procedures"[Mesh] OR neurosurgery OR craniotomy OR craniectomy) ) AND ( ("Air Travel"[Mesh] OR "air travel" OR flight OR aviation OR "aeromedical evacuation" OR "medical evacuation" OR air transport) ) AND ( (pneumocephalus OR "intracranial air" OR "Intracranial Pressure"[Mesh] OR ICP OR "intracranial hypertension" OR hypobaria OR hypoxia OR "barometric pressure") ) | 146 |
| **Embase (Elsevier)** | 24 July 2026 | ('brain injury'/exp OR 'brain injury' OR 'traumatic brain injury'/exp OR 'traumatic brain injury' OR tbi OR 'head injury'/exp OR 'head injury' OR 'neurosurgery'/exp OR 'neurosurgery' OR 'neurosurgical procedure'/exp OR 'neurosurgical procedure' OR 'craniotomy'/exp OR 'craniotomy' OR 'craniectomy'/exp OR 'craniectomy') AND ('aviation medicine'/exp OR 'aviation medicine' OR 'flight'/exp OR 'flight' OR 'aviation'/exp OR 'aviation' OR 'air travel'/exp OR 'air travel' OR 'commercial air travel' OR 'aeromedical evacuation'/exp OR 'aeromedical evacuation' OR 'medical evacuation'/exp OR 'medical evacuation' OR 'air transport'/exp OR 'air transport' OR 'helicopter transport'/exp OR 'helicopter transport') AND ('pneumocephalus'/exp OR 'pneumocephalus' OR 'intracranial air'/exp OR 'intracranial air' OR 'intracranial pressure'/exp OR 'intracranial pressure' OR icp OR 'intracranial hypertension'/exp OR 'intracranial hypertension' OR 'hypobaria'/exp OR 'hypobaria' OR 'hypoxia'/exp OR 'hypoxia' OR 'barometric pressure'/exp OR 'barometric pressure') | 384 |
| **Scopus** | 24 July 2026 | TITLE-ABS-KEY ( ( "traumatic brain injury" OR TBI OR "head injury" OR "intracranial injury" OR neurosurgery OR "neurosurgical procedure" OR craniotomy OR craniectomy ) ) AND TITLE-ABS-KEY ( ( "air travel" OR flight OR aviation OR "commercial air travel" OR "aeromedical evacuation" OR "medical evacuation" OR "air transport" OR "helicopter transport" ) ) AND TITLE-ABS-KEY ( ( pneumocephalus OR "intracranial air" OR "intracranial pressure" OR ICP OR "intracranial hypertension" OR hypobaria OR hypoxia OR "barometric pressure" ) ) | 129 |
| **ClinicalTrials.gov** | 24 July 2026 | ("traumatic brain injury" OR TBI OR "head injury" OR neurosurgery OR craniotomy OR craniectomy) AND (flight OR "air travel" OR aviation OR "air transport" OR "aeromedical evacuation" OR "medical evacuation" OR transport) AND (pneumocephalus OR "intracranial pressure" OR ICP OR "intracranial hypertension" OR hypoxia OR hypobaria OR "barometric pressure") | 75 |
